# Improved Progression-Free Survival for Bulky and Non-Bulky Advanced Stage Diffuse Large B-Cell Lymphoma With Consolidative Radiation Therapy: A Bi-Institutional Analysis

**DOI:** 10.7759/cureus.17107

**Published:** 2021-08-11

**Authors:** Yusef A Syed, Cecilia Jiang, Jeffrey Switchenko, Khadija Kirmani, Christopher Kelsey, Mohammad K Khan

**Affiliations:** 1 Department of Radiation Oncology, Winship Cancer Institute, Emory University, Atlanta, USA; 2 Department of Radiation Oncology, The University of Pennsylvania, Philadelphia, USA; 3 Department of Biostatistics and Bioinformatics, Winship Cancer Institute, Emory University, Atlanta, USA; 4 Department of Radiation Oncology, Lipscomb University, Nashville, USA; 5 Department of Radiation Oncology, Duke University Hospital, Durham, USA

**Keywords:** non-hodgkin’s, rituximab, r-chop, adjuvant, involved site, radiation therapy, imrt

## Abstract

Background

The role of consolidative radiation therapy (RT) for advanced-stage diffuse large B-cell lymphoma (DLBCL) is not fully established. A growing body of data suggests a role for consolidative RT in select stage III-IV DLBCL patients and emerging data from randomized studies further address the role of RT in advanced-stage patients initially presenting with bulky disease.

Methods

Patients with treatment-naive stage III-IV DLBCL treated at two institutions who achieved a clinically complete response to systemic therapy were included. Patients with either bulky or non-bulky disease were included, but those with the relapsed or refractory disease were excluded. Kaplan-Meier analysis was performed to determine the impact of consolidative RT. Univariate and multivariable analyses were performed using a Cox proportional hazards model.

Results

One hundred eighty-eight patients received systemic therapy consisting of rituximab, cyclophosphamide, doxorubicin, vincristine, and prednisone (R-CHOP; 79%), another rituximab-based regimen (9%), or chemotherapy alone (12%). Clinical response was assessed using conventional CT or PET-CT. Sixty-eight patients (36%) received consolidative RT (median dose 30 Gy). Consolidative RT conferred a 36.7% absolute benefit in five-year progression-free survival (PFS; 85.9% vs. 49.2%, log rank p < 0.0001), a 14.5% absolute benefit in five-year overall survival (OS; 87.4% vs. 72.9%, log rank p = 0.0134), and a 37.0% absolute benefit in five-year LC (91.9% vs. 54.9%, log rank p < 0.0001). On multivariable analysis, consolidative RT was associated with improved PFS (HR 0.23, 95% CI 0.10-0.52, p < 0.001) and LC (HR 0.20, 95% CI 0.07-0.59, p = 0.003). Patients receiving consolidative RT demonstrated significantly improved PFS for tumors measuring both <5 cm (log rank p = 0.0454) and ≥5 cm (log rank p = 0.0003).

Conclusions

For patients with stage III-IV DLBCL who achieve clinical complete response after systemic therapy, consolidative RT improves PFS for all patients, including those with the non-bulky disease. This benefit persists in the setting of rituximab-based systemic therapy.

## Introduction

Diffuse large B-cell lymphoma (DLBCL) is the most common aggressive non-Hodgkin lymphoma and a majority of these patients present with stage III-IV disease. Modern chemo-immunotherapy has contributed greatly to improving the survival rate and regimens consisting of rituximab, cyclophosphamide, doxorubicin, vincristine, and prednisone (R-CHOP) have become the standard of care. Recently, other regimens have been introduced to address aggressive pathological features such as double or triple hit mutational status [1]. Given that as many as two-thirds of patients present with advanced-stage disease and 10-year overall survival (OS) for stage II-IV patients receiving R-CHOP alone is reported at 43.5%; further efforts are warranted to improve outcomes for this patient subset [2,3].

In light of suboptimal patient outcomes, consolidative radiation therapy (RT) to sites of initially bulky disease or skeletal involvement is regarded as an option for treatment escalation upon the completion of chemo-immunotherapy, though strides made in systemic therapy have called into question the relative utility of RT. The RICOVER 60 trial showed a significant benefit in both event-free survival and overall survival with the addition of radiation therapy in elderly patients with bulky disease, including those with advanced disease [4]. For patients under 60 years of age, additional evidence for treatment comes from a growing body of retrospective literature from Duke and MD Anderson Cancer Center, among others, that suggests a benefit with consolidative RT [5-7].

At present, patients with stage III-IV DLBCL standardly undergo two to four cycles of rituximab-based systemic therapy with interim restaging and completion of six cycles for those who demonstrate a response. Consolidative RT, if elected, is administered thereafter, though prospective data supporting its use are still forthcoming. Nonetheless, prior studies have shown that a majority of patients fail locally, particularly at sites of initially bulky disease, even after reaching clinical complete response [6,8].

The aforementioned uncertainty with regards to the role of RT in advanced stage DLBCL has resulted in widely divergent practice patterns. Some institutions have elected to treat select stage III-IV patients, in particular those who present with bulky disease, exhibit a partial response to chemotherapy, or demonstrate limited skeletal involvement at initial presentation. Still, others resort to systemic therapy alone. As the exact role for consolidative radiotherapy in advanced stage DLBCL has yet to be fully elucidated, a subset of patients may be forgoing a potentially beneficial therapy at present. The data presented here build upon previously published work to further examine the potential benefits offered by consolidative RT.

The article was previously published in the ResearchSquare preprint server (https://www.researchsquare.com/article/rs-137697/v1).

## Materials and methods

This bi-institutional retrospective study was approved by the Institutional Review Boards at Emory University and Duke University. Inclusion criteria consisted of treatment-naive patients diagnosed with stage III-IV DLBCL between April 1999 and January 2011 who had a documented clinical complete response to systemic therapy (cCR). Patients with either bulky or non-bulky disease were included; however, patients with involvement of the central nervous system and those with the relapsed or refractory disease were excluded. The staging was determined based on the Ann Arbor classification system [9]. Although the diagnostic modalities used for staging varied by the patient, the components generally included computed tomography (CT) of the chest, abdomen and pelvis, bone marrow aspirate, and biopsy, with positron emission tomography (PET) of the body used in a subset of cases. Molecular profiling was not routinely completed and thus was excluded from this analysis. An International Prognostic Index (IPI) was also calculated for each patient and used as a prognostic variable [10]. The bulky disease was defined as that measuring 5 cm or larger in maximal diameter. This was used instead of the conventional 7.5 cm threshold based upon prior work finding 5 cm to be a meaningful cut point [11]. Furthermore, a central aim of this work is to establish a clinical benefit for the use of consolidative RT at a lower threshold of bulky disease. 

Patients were treated with a range of systemic therapy regimens, including rituximab combined with cyclophosphamide, doxorubicin, vincristine, and prednisone (R-CHOP). The response was assessed based on CT or PET imaging and a cCR to systemic therapy as documented in imaging reports was necessary for inclusion in this study. The response was assessed using the International Harmonizing Project in Lymphoma criteria [12,13]. Those with an incomplete response or refractory disease were excluded. Following cCR, a subset of patients was additionally treated with consolidative RT at the discretion of the treating medical and radiation oncologists. Patients were treated at either the Winship Cancer Institute at Emory University (Atlanta, GA) or the Duke Cancer Institute (Durham, NC). Patients were treated using 3D conformal RT or intensity-modulated RT (IMRT) techniques delivered by conventional linear accelerators at both sites. Radiation therapy was generally given using modern principles of delivering treatment to the original sites of disease with a small margin, consistent with modern involved site RT, though the RT protocol was not standardized and was not a focus of this study. Clinical endpoints were measured from the date of diagnosis, as is customary for retrospective studies of this nature in which the timing and duration of treatments vary between patients.

Statistical methods

Patients were divided into a chemotherapy-only cohort or chemotherapy plus RT cohort for analysis. Patient demographics, along with IPI score and bone or extranodal involvement were recorded. Numerical variables such as age at diagnosis were reported as median values with ranges. A chi-square test or Fisher’s exact test were used to compare differences in categorical variables between cohorts, whereas differences in numerical covariates were compared with ANOVA. A p-value of less than 0.05 was considered statistically significant.

Patients were subsequently followed with disease recurrences documented to assess progression-free survival (PFS). PFS at each time point was defined as an absence of radiographically or clinically apparent disease, measured from the time of diagnosis to any failure, or death. Those patients who did not experience treatment failure were censored at the last follow-up. Other endpoints of interest included OS, defined from the date of diagnosis to the date of death or last follow-up, and local control (LC), defined from the date of diagnosis to the date of in-field failure or failure at an initially presenting site of disease. Patients who survived or experienced no disease progression were censored at the date of the last follow-up.

Kaplan-Meier plots were generated for PFS, OS, and LC, and a log-rank test was performed to assess for differences between cohorts with respect to the endpoints. One-year, two-year, and five-year OS, PFS, and LC rates with 95% confidence intervals (CI) were also calculated separately by cohort. Additionally, univariate and multivariable Cox proportional hazard models were performed to assess for the effect of selected categorical covariates on PFS, OS, and LC. All statistical analysis was performed using SAS version 9.4 (SAS, Cary, NC).

## Results

A total of 287 patients received systemic therapy for confirmed DLBCL. After excluding patients who did not have stage III-IV disease at the time of treatment or did not demonstrate a complete response to systemic therapy, 188 patients meeting inclusion criteria remained and comprised the study population. Of these, 79 patients were treated at Duke and 109 patients were treated at Emory. Stage III DLBCL comprised 36% of patients compared to 64% with stage IV disease. R-CHOP was administered to 79% of patients, while 9% received another rituximab-based regimen, and 12% were treated without rituximab. The staging was completed using PET-CT in 76 cases (40%), with conventional CT alone used in the remaining cases. 

In total, 68 patients (36%) received consolidative radiotherapy after systemic therapy while 120 patients (64%) received systemic therapy alone. Patient characteristics divided by cohort are depicted in Table 1. For those who received consolidative RT, patients were treated to a median of 30 Gy (range 12 Gy to 40.8 Gy) at 1.5 to 3 Gy per fraction. Patients receiving consolidative RT were more likely to have the bulky disease (69% of radiation patients had bulky disease compared to 53% of non-radiation patients, p = 0.045), though no other significant differences between cohorts were noted. Patients treated at Duke were more likely to receive RT than those treated at Emory (48% vs. 28%, p = 0.004).

**Table 1:** Patient demographics. ECOG: Eastern Cooperative Oncology Group performance status, IPI: international prognostic index, LDH: serum lactate dehydrogenase, BM: bone marrow, R-CHOP: rituximab, cyclophosphamide, doxorubicin, vincristine, prednisone, R others: any rituximab-containing regimen other than R-CHOP.

Covariate	Value	Radiation		P-value
		No (N=120) N (%)	Yes (N=68) N (%)	
Study site	Emory	79 (65.8%)	30 (44.2%)	0.004
	Duke	41 (34.2%)	38 (55.8%)	
Gender	Female	59 (49.2%)	31 (45.6%)	0.637
	Male	61 (50.8%)	37 (54.4%)	
ECOG	0	62 (52.1%)	42 (62.7%)	0.343
	1	52 (43.7%)	22 (32.8%)	
	2/3	5 (4.2%)	3 (4.5%)	
IPI	1	19 (15.8%)	9 (13.2%)	0.087
	2	35 (29.2%)	26 (38.2%)	
	3	39 (32.5%)	27 (39.7%)	
	4/5	27 (22.5%)	6 (8.8%)	
Stage	3	45 (37.5%)	23 (33.8%)	0.614
	4	75 (62.5%)	45 (66.2%)	
Size of the largest site	<5 cm	46 (47.4%)	18 (31.0%)	0.045
	>5 cm	51 (52.6%)	40 (69.0%)	
B symptoms	No	67 (55.8%)	45 (66.2%)	0.165
	Yes	53 (44.2%)	23 (33.8%)	
LDH	Normal	26 (27.4%)	14 (25.5%)	0.798
	Above normal	69 (72.6%)	41 (74.6%)	
Extranodal sites	No	23 (19.5%)	17 (25.0%)	0.379
	Yes	95 (80.5%)	51 (75.0%)	
BM involvement	No	87 (75.0%)	51 (78.5%)	0.600
	Yes	29 (25.0%)	14 (21.5%)	
Chemotherapy regimen	R-CHOP	95 (79.2%)	53 (77.9%)	0.079
	R others	14 (11.7%)	3 (4.4%)	
	No R	11 (9.2%)	12 (17.7%)	
Number of cycles	3-4	17 (14.2%)	17 (25.0%)	0.064
	>4	103 (85.8%)	51 (75.0%)	
Age at diagnosis (years)	Mean	58.8	56.8	0.381
	Median	59.6	59.0	

**Figure 1:**
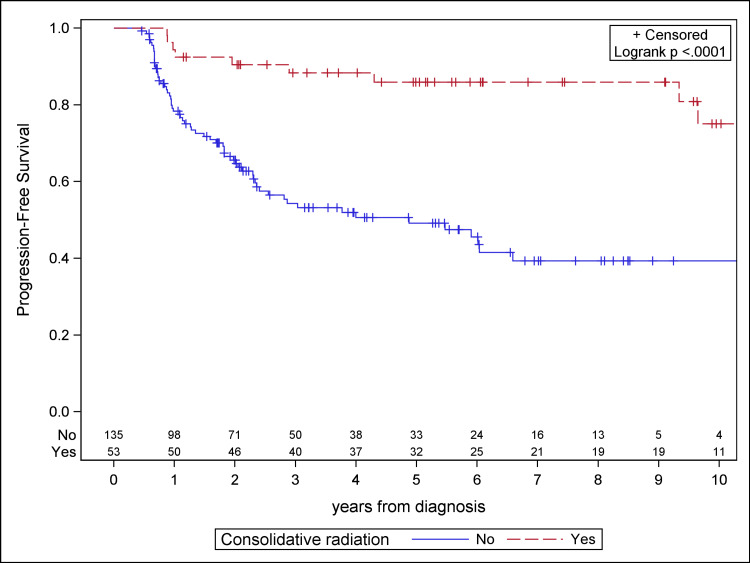
Kaplan-Meier plot for progression-free survival for the study cohort.

**Figure 2:**
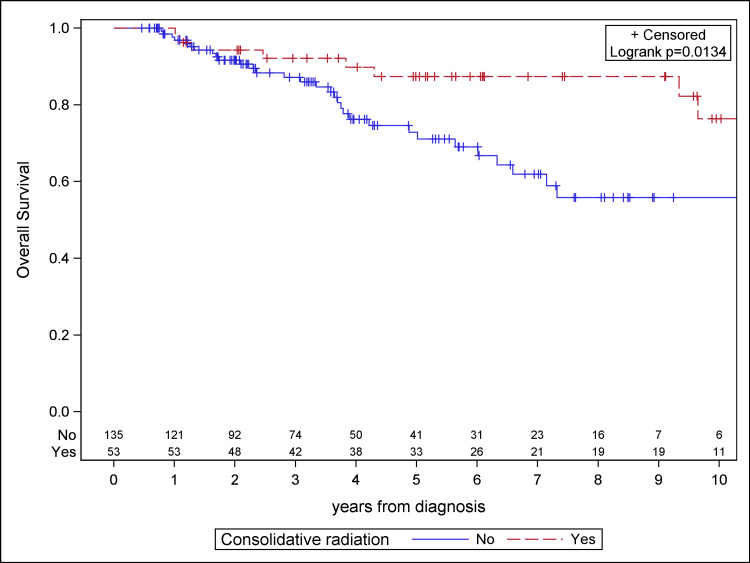
Kaplan-Meier plot for overall survival for the study cohort.

**Figure 3:**
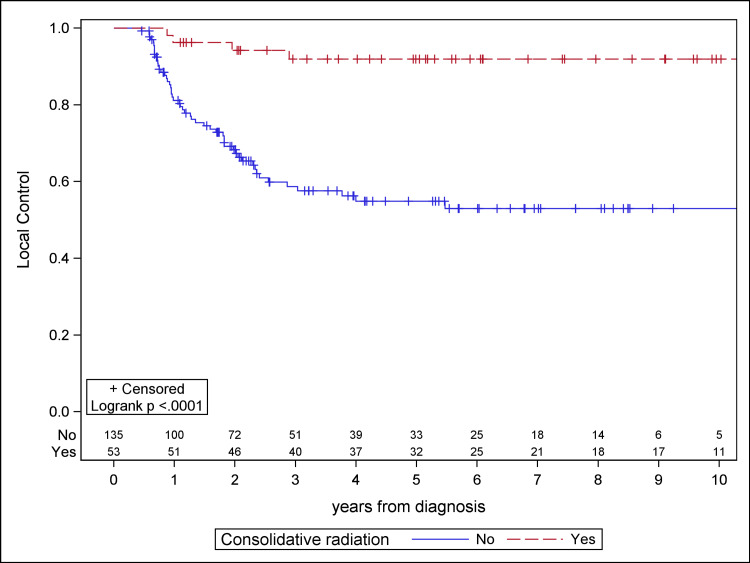
Kaplan-Meier plot for local control for the study cohort.

The median follow-up for the study population was 4.1 years. Patients receiving consolidative RT demonstrated significantly improved PFS, OS, and LC compared to the chemotherapy cohort on Kaplan-Meier analysis (Figures 1-3). Median PFS was 4.9 years for the chemotherapy-only cohort and not reached for the consolidative RT cohort. Median OS was 6.6 years for the chemotherapy-only cohort and was not reached for the consolidative RT cohort. Median time to local failure was not reached for either cohort. With the addition of radiation, there was a 36.7% absolute benefit in five-year PFS (85.9% vs. 49.2%, log-rank p < 0.0001), a 14.5% absolute benefit in five-year OS (87.4% vs. 72.9%, log-rank p = 0.0134), and a 37.0% absolute benefit in five-year LC (91.9% vs. 54.9%, log-rank p < 0.0001).

On univariate analysis, the use of RT was associated with improved PFS (HR 0.22, p < 0.001; Table 2). Four variables were associated with OS: use of RT vs. no RT (HR 0.38, p = 0.017), IPI 4/5 vs IPI 1 (HR 3.75, p = 0.043), R-other vs. R-CHOP (HR 2.81, p = 0.015), and bone marrow involvement (HR 2.06, p = 0.033). On multivariable analysis, the association between RT and PFS persisted (HR 0.23, 95% CI 0.10-0.52, p < 0.001) when adjusting for differences in IPI, tumor size, systemic therapy regimen, and extranodal involvement. However, the association between consolidative RT and OS was reduced when adjusting for these same co-variables (HR 0.55, 95% CI 0.21-1.42, p = 0.216). The association between consolidative RT and LC persisted on multivariable analysis (HR 0.20, 95% CI 0.07-0.59, p = 0.003) when adjusting for study site, size of the largest site, and the number of cycles, which were all significant on univariate analysis.

**Table 2:** Univariate and multivariable analyses for PFS and OS, and LC. IPI: International Prognostic Index; Y: yes; N: no; No R: chemotherapy alone; R others: rituximab-containing regimen other than R-CHOP.

	Univariate analysis		Multivariable analysis		
	HR	p-value	HR	95% CI	p-value
Progression-free survival					
Radiation (Y vs. N)	0.22	<0.001	0.23	0.10-0.52	<0.001
IPI 4/5 vs. 1	1.29	0.517	0.81	0.30-2.17	0.679
IPI 3 vs. 1	1.21	0.596	1.05	0.44-2.51	0.91
IPI 2 vs. 1	0.87	0.71	0.69	0.27-1.77	0.438
Tumor size >5 vs. <5 (cm)	1.43	0.217	1.80	1.01-3.22	0.048
No R vs. R-CHOP	0.62	0.216	0.85	0.35-2.06	0.724
R others vs. R-CHOP	1.85	0.087	1.45	0.61-3.42	0.397
Extranodal sites (Y vs. N)	1.52	0.176	1.61	0.74-3.49	0.231
Overall survival					
Radiation (Y vs. N)	0.38	0.017	0.55	0.21-1.42	0.216
IPI 4/5 vs. 1	3.75	0.043	2.34	0.56-9.82	0.245
IPI 3 vs. 1	2.80	0.103	1.79	0.47-6.79	0.395
IPI 2 vs. 1	1.43	0.589	1.09	0.26-4.55	0.909
Tumor size >5 vs. <5 (cm)	0.84	0.627	0.92	0.43-1.94	0.816
No R vs. R-CHOP	0.57	0.276	0.50	0.15-1.66	0.26
R others vs. R-CHOP	2.81	0.015	1.56	0.55-4.38	0.402
Extranodal sites (Y vs. N)	1.54	0.299	0.91	0.35-2.38	0.853
Local control					
Radiation (Y vs. N)	0.14	<0.001	0.20	0.07-0.59	0.003
Tumor size >5 vs. <5 (cm)	2.25	0.028	2.92	1.40-6.08	0.004
Number of cycles, >4 vs. 3-4	3.62	0.013	1.98	0.56-6.99	0.291

**Figure 4:**
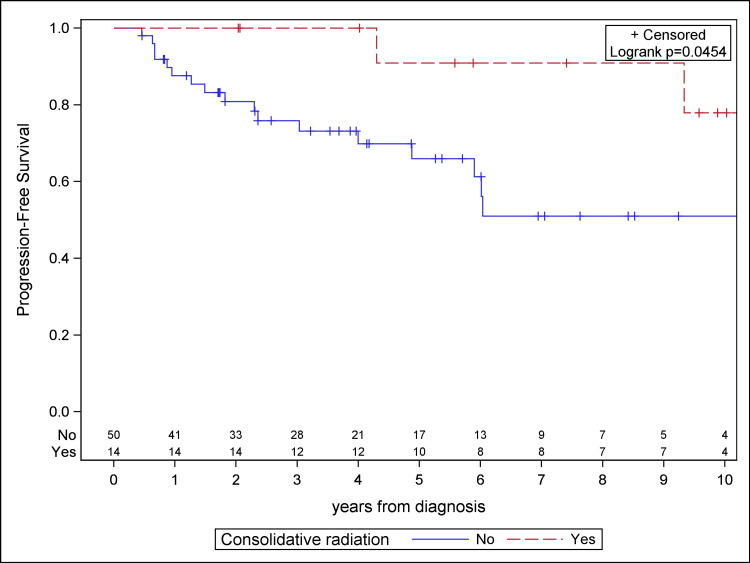
Kaplan-Meier plot for progression-free survival for patients with tumors <5 cm in maximal diameter.

**Figure 5:**
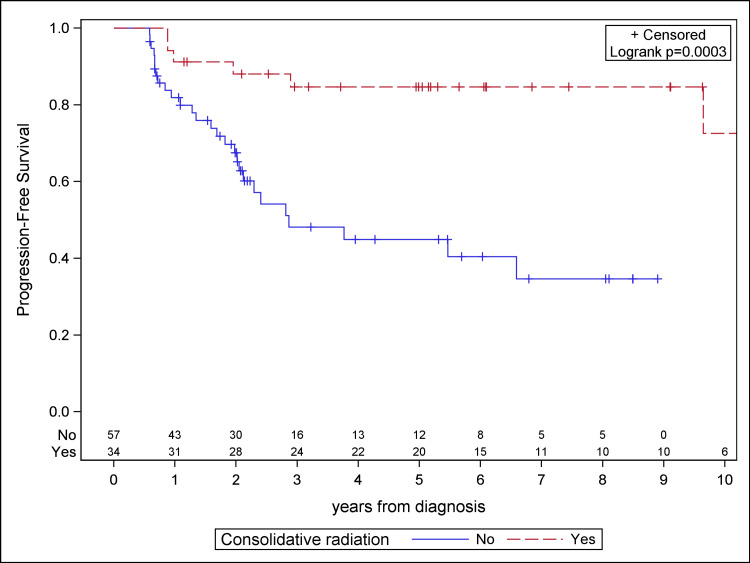
Kaplan-Meier plot for progression-free survival for patients with tumors ≥5 cm in maximal diameter.

The impact of consolidative radiation with respect to tumor size was explored by prospectively setting a threshold maximal tumor diameter of 5 cm for the largest mass observed on initial staging imaging. Patients receiving consolidative RT demonstrated significantly improved PFS for disease measuring both <5 cm (log-rank, p = 0.0454) and ≥5 cm (log-rank p = 0.0003) in maximal diameter (Figures 4-5). No difference in OS was observed with respect to tumor size at a threshold of 5 cm. Of note, no patient with tumor size <5 cm receiving consolidative RT experienced a local failure. The multivariable analysis also showed tumor size ≥5 cm vs. <5 cm to be associated with worse PFS (HR 1.80, 95% CI 1.01-3.22, p = 0.048).

## Discussion

The addition of consolidative RT for advanced-stage DLBCL patients who demonstrate complete response after chemotherapy is associated with a statistically significant improvement in overall survival and progression-free survival and local control when pooling data from two academic medical centers. This analysis provides an additional basis for the utilization of consolidative RT in these patients and contributes to a growing body of prospective and retrospective work. These combined data corroborate earlier retrospective studies, which collectively document improvements in overall survival, event-free survival, progression-free survival, and local control with consolidative RT. Forthcoming randomized data are expected to further elucidate the role of RT for this group. Historically, stage III-IV patients have often been aggregated with stage I-II patients, potentially diluting any measurable benefit associated with consolidative RT. Thus, the true role of consolidative RT in this population is evolving.

Phan et al. previously reported statistically significant improvements in both OS and PFS at five years for a large cohort of DLBCL patients, consistent with the results presented here [7]. The majority of patients in the Phan cohort presented with stage III-IV disease (279 from a total of 469), however, RT was delivered to only 39 (14%) advanced-stage patients in their analysis as compared with 53 (28%) of patients in our cohort. OS and PFS were both significantly improved for advanced-stage patients. They report five-year OS and PFS after RT of 89% and 76%, respectively, as compared with our figures of 87% and 86%, respectively, at the same time point.

Dorth et al. report excellent in-field control and event-free survival for stage III-IV patients at five years with consolidative RT (92% and 85%, respectively) [5]. They reported a five-year OS survival of 85% for RT patients and demonstrated a trend towards improvement but fell short of achieving statistical significance (78% for the no-RT subset, p = 0.15). The addition of advanced stage Emory patients bolstered the case for an OS advantage though failed to meet criteria for significance on multivariable analysis, likely due to confounding factors related to patient selection, tumor size, and sample size. Nonetheless, our combined Kaplan-Meier analysis demonstrated a clinically meaningful 14.5% improvement at five years with the pooled results. Importantly, our combined analysis found a persistent benefit with respect to local control, paralleling previously published results and establishing a framework for the observed progression-free survival benefit.

The definition of bulky disease from the standpoint of consolidation is a topic of ongoing debate, but the existing body of literature points to tumor size affecting treatment outcomes. Post-hoc analysis of the MInT study found cut-off sizes of 6 cm and 10 cm corresponded to statistically significant differences in OS and EFS, respectively, among patients treated with R-CHOP and consolidative RT [14,15]. Additionally, prior work by our group has demonstrated a 5 cm cut-point to be meaningful with respect to local control [11]. This analysis builds upon these prior findings by demonstrating a PFS benefit that persists with a 5 cm cut-off point. Thus, the results presented here support extending consolidative RT to a larger subset of advanced-stage DLBCL patients.

The advent of rituximab substantially improved survival outcomes for lymphoma patients, firmly establishing R-CHOP as the standard of care for DLBCL [2,15-17]. This has led some to question the utility of consolidative RT, particularly as it exposes patients to a separate array of potential toxicities. Thus, the therapeutic effect of consolidative RT, specifically for patients receiving R-CHOP, was of particular interest in our analysis as well as in the other studies described here. Fully 84% percent of MDACC patients and 88% of patients in the Emory/Duke combined cohort received a complete or partial course of rituximab-based systemic therapy. Results from these three centers demonstrate a substantial and clinically meaningful benefit for consolidative RT in patients treated with rituximab, corroborating results of a recent NCCN outcomes database analysis for DLBCL patients across all stages [18].

The results presented here complement prior retrospective studies and support further investigations of consolidative RT in stage III-IV DLBCL. The RICOVER-60 trial included DLBCL patients across all stages and primarily evaluated the addition of rituximab to six versus eight cycles of CHOP-14, with consolidative RT, delivered to sites of initially bulky (≥7.5 cm) or extra lymphatic disease. The impact of RT was later examined by means of a protocol amendment that created a no-RT arm that was not included in the initial randomization. The intent-to-treat analysis showed improved event-free survival and a trend towards improved PFS and OS (the latter two were significantly improved in the per-protocol analysis). The ongoing UNFOLDER trial, published in abstract form, randomized a similar group of patients to two R-CHOP regimens with a second randomization to consolidative RT to bulky and extra-nodal disease [19]. Of note, the no-RT arm was closed at interim analysis. Additionally, results from the OPTIMAL>60 trial were published in abstract form and suggest that for elderly patients RT can be spared in bulky disease that is PET-negative after chemotherapy, though patients with the persistent disease received RT per protocol [20].

Aside from radiation therapy, other consolidation strategies have been explored for advanced-stage DLBCL. The randomized, phase II GELA/LYSA trial sought to evaluate the efficacy of two rituximab-based induction regimens along with a PET-driven consolidation regimen [21]. According to this protocol, patients with negative PET-CT after completion of four cycles of induction therapy (PET4) were treated based upon an interim PET-CT completed after two induction cycles (PET2); patients with negative PET2 received consolidative immunochemotherapy while those with positive PET2 received chemotherapy and autologous stem cell transplant (ASCT). For patients treated with induction R-CHOP who exhibited cCR, four-year results show a PFS of 82.2% and OS of 87.2%. These figures compare favorably with results presented here for consolidative RT (85.9% and 87.4%, respectively, at five years), suggesting that radiation therapy may be a reasonable alternative to additional chemo-immunotherapy or ASCT.

One shortcoming of our work is that RT dosing varied significantly across patients in our study cohort with a median dose of 30 Gy, which is lower than doses utilized in many previous large randomized trials of consolidative IFRT in stage I-II non-Hodgkin lymphoma; SWOG 8736 used 40-55 Gy, while LHN 93-1 and 93-4 used 40 Gy, though ECOG 1484 did use 30 Gy [22-25]. These trials were conducted in the pre-rituximab era and a subsequent meta-analysis incorporating selected data from these four trials revealed a PFS improvement with IFRT but failed to demonstrate improvement in OS. This meta-analysis was limited by heterogeneity in the pooled dataset, however [26]. For patients with advanced disease who respond well to systemic therapy, it has been suggested that lower RT doses may, in fact, be preferred since these patients are likely to have received longer courses of chemotherapy and RT will be delivered to larger volumes [27]. As such, 30 Gy is regarded as a standard dose at this time.

Other shortcomings include the non-standardization of imaging in response assessment and the age of our dataset. It is now established that PET-CT is the gold standard in the staging of aggressive NHL and has higher specificity, accuracy, and positive predictive value in detecting residual disease after systemic therapy than CT alone, though this practice was not established when some of our earlier patients were diagnosed [28,29]. While PET-CT was utilized in many cases, this was not a strict requirement for patients in this combined dataset. As such, we were not able to derive meaningful associations related to specific imaging findings, such as Deauville (5 points) score or SUV values as demonstrated previously [11]. Finally, concerning our timeline, advances in radiation oncology, including image guidance and volumetric dose planning, have improved the quality of care since the time that our earliest patients received treatment.

## Conclusions

For patients with stage III-IV DLBCL who achieve clinically complete response after systemic therapy, this retrospective, bi-institutional analysis demonstrates that consolidative RT improves progression-free survival for all patients, including those with the non-bulky disease. This benefit persists in the setting of rituximab-based systemic therapy.
